# Retraction: MicroRNA-497 suppresses renal cell carcinoma by targeting VEGFR-2 in ACHN cells

**DOI:** 10.1042/BSR20170270_RET

**Published:** 2025-08-18

This article is being retracted from *Bioscience Reports* at the request of the Editor-in-Chief and the Editorial Board. This follows the corresponding author contacting the Journal to request a correction of Figure 3B. They had identified three duplications: between the Figure 3A Control and the Figure 3B miR-497 inhibitor panels, between sections of the Figure 3B Control and Mimics NC panels, and between sections of the Figure 3B Mimics NC and Inhibitor NC panels. These duplications have been confirmed by the Editorial Board. The author explained that the duplications arose due to inconsistencies in file naming and storage during manuscript preparation. However, they were unable to provide the original raw data requested, and aside from the corresponding author, none of the co-authors could be reached for comment. The Editorial Board has therefore lost confidence in the validity of the data and stands by the decision to retract. The corresponding author disagrees to the retraction.

